# Surgical complications and their impact on patients’ psychosocial well-being: a systematic review and meta-analysis

**DOI:** 10.1136/bmjopen-2014-007224

**Published:** 2016-02-16

**Authors:** Anna Pinto, Omar Faiz, Rachel Davis, Alex Almoudaris, Charles Vincent

**Affiliations:** 1 Division of Surgery, Department of Surgery & Cancer, Imperial College London, London, UK; 2 Department of Experimental Psychology, Oxford University, Oxford, UK

**Keywords:** SURGERY

## Abstract

**Objective:**

Surgical complications may affect patients psychologically due to challenges such as prolonged recovery or long-lasting disability. Psychological distress could further delay patients’ recovery as stress delays wound healing and compromises immunity. This review investigates whether surgical complications adversely affect patients’ postoperative well-being and the duration of this impact.

**Methods:**

The primary data sources were ‘PsychINFO’, ‘EMBASE’ and ‘MEDLINE’ through OvidSP (year 2000 to May 2012). The reference lists of eligible articles were also reviewed. Studies were eligible if they measured the association of complications after major surgery from 4 surgical specialties (ie, cardiac, thoracic, gastrointestinal and vascular) with adult patients’ postoperative psychosocial outcomes using validated tools or psychological assessment. 13 605 articles were identified. 2 researchers independently extracted information from the included articles on study aims, participants’ characteristics, study design, surgical procedures, surgical complications, psychosocial outcomes and findings. The studies were synthesised narratively (ie, using text). Supplementary meta-analyses of the impact of surgical complications on psychosocial outcomes were also conducted.

**Results:**

50 studies were included in the narrative synthesis. Two-thirds of the studies found that patients who suffered surgical complications had significantly worse postoperative psychosocial outcomes even after controlling for preoperative psychosocial outcomes, clinical and demographic factors. Half of the studies with significant findings reported significant adverse effects of complications on patient psychosocial outcomes at 12 months (or more) postsurgery. 3 supplementary meta-analyses were completed, 1 on anxiety (including 2 studies) and 2 on physical and mental quality of life (including 3 studies). The latter indicated statistically significantly lower physical and mental quality of life (p<0.001) for patients who suffered surgical complications.

**Conclusions:**

Surgical complications appear to be a significant and often long-term predictor of patient postoperative psychosocial outcomes. The results highlight the importance of attending to patients’ psychological needs in the aftermath of surgical complications.

**Strengths and limitations of this study**

- This is, to our knowledge, the first systematic review of the literature assessing the impact of surgical complications on patients’ psychosocial well-being.
- The validity of the findings is increased by the fact that only studies that used validated self-report measures for the assessment of patients’ well-being were included in the review, as well as by the use of a very comprehensive search strategy for the identification of relevant literature.
- Caution should be taken when interpreting these findings to other specialties as the review was limited in four surgical specialties.
- A limitation of this review was the very small number of studies with sufficient data for the quantitative synthesis, which did not also permit certain types of sensitivity analyses such as by surgical specialty or type of surgery.

## Introduction

Surgical complications pose significant challenges for surgical patients. Complications may vary from very minor events that can be resolved relatively quickly without the need for pharmacological treatment or other intervention, to more serious events which can be life threatening, require multiple interventions (eg, return to theatre), delay patient's discharge and may lead to multiorgan failure or even death.^1^ A recent review of the literature found that postoperative complications contribute to increased mortality, length of stay and an increased level of care at discharge.^2^

Other than the complications’ impact on patients’ postoperative recovery, they may also affect patients psychologically. They may contribute to the experience of psychological distress such as depression or anxiety due to the challenges that are inherent to them in terms of prolonged recovery or long-lasting disability (eg, severe postoperative pain, permanent disfigurement). An early study found that patients who experienced serious adverse events after surgery reported higher levels of distress than people who had experienced serious accidents or bereavements and psychosocial adjustment worse than in patients with serious medical conditions.^3^ Moreover, the authors of an interview study on patients’ experiences of cardiothoracic surgery reported that a small number of patients who had a long and complicated postoperative hospital stay expressed intense feelings of hopelessness and depression.^4^ Psychological distress resulting from the experience of surgical complications could further delay patients’ recovery from surgery as increased levels of stress delay wound healing^5^ ^6^ and compromise immunity.^7–9^

This review aims to critically review and synthesise the existing literature on the impact of surgical complications on adult surgical patients’ psychosocial well-being and to estimate the duration of this impact. For the purpose of this review, psychosocial well-being was defined quite broadly including psychosocial outcomes of relevance to surgery such as anxiety, depression, quality of life (QoL) and post-traumatic stress. Quantitative studies which assessed the association of surgical complications with adult patients’ psycho-social outcomes post-surgery were therefore reviewed. Our hypothesis was that the occurrence of surgical complications adversely affects patient psychosocial outcomes. Therefore, this systematic review aims to examine whether surgical complications impact adversely on patient psychosocial outcomes and the duration of this impact.

## Methods

### Search strategy

The following databases were searched through OvidSP: ‘PsychINFO’ (1967 to 25 May 2012), ‘EMBASE’ (1947 to 25 May 2012) and ‘MEDLINE’ (1948 to 25 May 2012). A search strategy was developed specific to each database. The three facets of the search strategy were:

- Adult surgical patients
  - Terms such as patients, inpatients, outpatients, men and women were used for this facet.
- Patient psychosocial outcomes
  - A broad definition of psychosocial outcomes was considered for the purposes of this systemic review including search terms for anxiety, depression, QoL and post-traumatic stress.^10^ Two generic terms were also used, that is, well-being and emotions. The search did not include specific measures, instead it included terms for the outcomes specified above.
- Surgical complications
  - Surgical complications were defined as any adverse event in relation to the surgical procedure including search terms for complications (eg, adverse events, untoward incidents) and terms about the surgical setting (eg, surgical, postoperative).

Each of the facets was expanded into a list of search terms truncated and combined with each other using Boolean operators, and also by mapping those to their relevant MeSH headings and subheadings in each database (through explosion of each MeSH heading). The search was restricted to titles and abstracts, and the results were limited to studies that used human participants and were written in English. The search strategies are presented in online supplementary material 1. Database searching was complemented by reviewing the reference lists of eligible articles.

### Eligibility criteria

Studies were included in the review if they met the following criteria:

- Any quantitative study that measured the association of surgical complications with adult patients’ psychosocial outcomes after surgery, either as a primary or secondary aim. Studies that measured surgical complications and psychosocial outcomes but not their association were not included as a primary analysis of reported data was beyond the scope of this review. Moreover, specific types of complications were not predefined as this review was interested in the impact of any surgical complications on patients’ well-being.
- Psychosocial outcomes were measured with validated self-report tools or psychological assessment.
- Studies that reported surgical complications after cardiac, thoracic, gastrointestinal or vascular surgery, where complications are more likely to occur.^11^ Studies of neuropsychological complications (eg, delirium) and studies of transplantation procedures were excluded.

Conference proceedings, non-empirical data and articles that were published before the year 2000 or with the majority of their participants recruited before the year 2000 were excluded. This current approach in the selection of literature was expected to reduce bias resulting from studies of out-dated surgical practices.

### Study selection

A total of 50% of the abstracts were reviewed independently by two researchers (AP and RD) and disagreements were resolved by consensus. The remaining half of the retrieved abstracts were reviewed by the primary researcher (AP) based on the consensus that was achieved for the first half. After excluding ineligible articles at abstract and title level, the remaining articles were assessed in full text. The eligibility criteria were applied again on each article. Reasons for exclusion were coded. Articles for which there was uncertainty were discussed between the primary researcher (AP), a researcher with background in psychology (RD) and a researcher with background in surgery (AA). Any disagreements were resolved by consensus.

### Data extraction and quality assessment

The primary researcher (AP) and a researcher with a background in surgery (AA) independently extracted data from 20 articles, which they reviewed for any disagreements. Disagreements were resolved by consensus or referral to a third senior researcher (OF). Data were extracted from the remaining articles by the primary researcher and were later checked by the second reviewer (AA). A total of 10 authors were contacted by email to provide information that was not included in the manuscripts. Three articles were excluded from the analysis because their authors did not respond to our requests for further information. Information was extracted from each article on study aims, participants’ characteristics, study design, surgical procedures, surgical complications (ie, types, definitions and method of recording, where available), psychosocial outcomes (ie, scales, and time points of measurement), and the association of psychosocial outcomes with surgical complications. The latter included any reported findings on the association of surgical complications with the psychosocial outcomes, including both overall scale and subscale scores where available.

The quality of the included studies was assessed with the Newcastle Ottawa Scales (NOS).^12^ The scales were modified in order to reflect the research questions of the review and to also incorporate the assessment of cross-sectional studies.

### Data synthesis

The included studies were first synthesised narratively (ie, using words and text). In order to quantify the degree of the impact of surgical complications on psychosocial outcomes, quantitative procedures were also used. A meta-analysis was conducted on each extracted psychosocial outcome using Review Manager (V.5.2).^13^ I^2^ was used to calculate the heterogeneity present in the meta-analyses. Heterogeneity was considered low when it was below 25% and high above 50%.^14^ A random-effects approach was chosen, as a degree of heterogeneity between studies should always be assumed in social sciences.^15^ Where multiple assessments were conducted in one single study, only the one furthest from the participants’ surgery was included in the meta-analysis.

## Results

**Figure 1:**
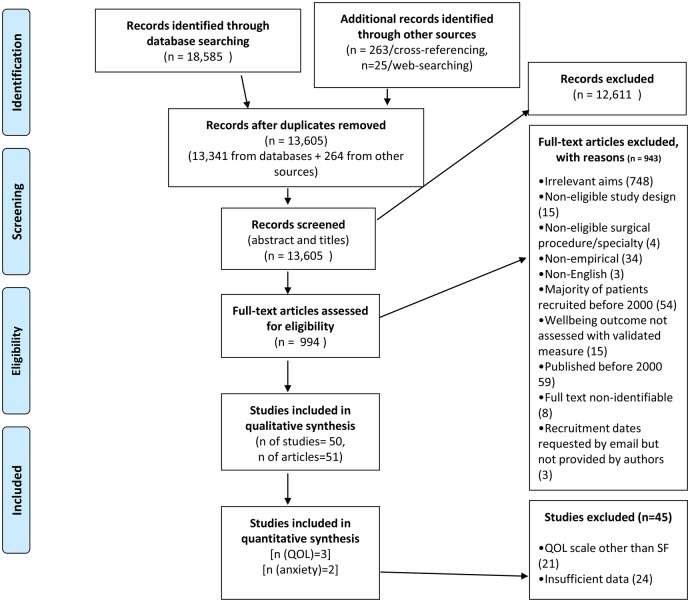
PRISMA diagram. SF, Short Form Health Survey; QOL, quality of life.

In total, 18 585 articles were retrieved in total across the three databases. After removing duplicate references, a total of 13 605 papers were reviewed at abstract and title level. Nine hundred and ninety-four articles remained to be assessed in full text. A total of 51 articles (50 studies) were eligible for inclusion in the final stage of the review (see figure 1).

### Study characteristics

Details of the included studies are presented in tables 1–3. A total of 28 studies were conducted in Europe, 14 in the USA, 3 in Australia, 2 in Turkey, 1 in Egypt, 1 in Japan and 1 in Taiwan. There were 29 studies in gastrointestinal,^16–44^ 17 in cardiothoracic^45–62^ and 4 in vascular surgery.^63–66^ The majority of the included studies (40 studies) assessed major procedures. The most common indications for surgery were heart conditions, followed by different types of cancer. Twenty-three studies examined the association between surgical complications and patients’ well-being as a primary research aim.^17^ ^19^ ^28^ ^30–38^ ^43^ ^47^ ^48^ ^50–53^ ^55^ ^62^ ^64^ ^66^ The remaining examined this relationship as part of an exploration of the association of different clinical factors with patients’ postoperative well-being. The majority of the studies were cohort studies. There were four case–control and 20 cross-sectional studies. The majority of the studies were prospective, including baseline measures of psychosocial outcomes.

**Table 1:** Key characteristics of gastrointestinal surgery studies (n=29)

First author's name	Year	Country	Primary or Secondary aim	Sample (N=number of patients in analysis/eligible patients, Nt(i)=sample size per time point, Nc=patients with complications, N1=cases vs N2=controls)	Patient inclusion criteria	Study design	Type of surgery	Surgical complications/method of recording	Psychosocial outcome/time points/measurement tool	Significant association of surgical complications with patients’ well-being (yes/no/confounding)	Types of complications and time points of significant effects	Quality assessment score (out of 8)
Anthony	2003	US	Secondary	Nt1=71/? Nt2=63 Nc=16	Colorectal cancer, male patients who underwent open surgical therapy	Observational, cohort, prospective	Open surgical therapy for colorectal cancer	*Morbidity* was defined as any event that resulted in the need for additional therapy or readmission to the hospital within 30 days of initial discharge/method not specified	QoL/at time of diagnosis and 12 months after surgery/FACT-C	Yes*	Any complications/12 months postsurgery	6
Avery	2006	UK	Primary	N=139/162 Nc=37	Patients with oesophageal or gastric cancer who underwent upper gastrointestinal surgical treatment	Observational, cross-sectional	Upper gastrointestinal surgical treatment for oesophageal or gastric cancer	A major complication was defined as reoperation, readmission to the high dependency or intensive care unit, readmission to the hospital within 30 days of operation, or death within 30 days of operation or later if the patient did not leave the hospital/method not specified	QOL/39.6 days after treatment (range 6–105)/EORTC QLQ-C30	Yes	Any complications/39.6 days after treatment (range 6–105)	5
Bitzer	2008	Germany	Secondary	Nt1=151/205 Nt2=130 (86.1%) Nc(complaints)=49 Nc(wound infection)=5 Nc(seroma)=13 Nc(pneumonia)=1 Nc(other)=28	Patients undergoing cholecystectomy	Observational, cohort, prospective	Cholecystectomy	Retrospective list: any complaint, wound infection, seroma, pneumonia, other complaints/patient reports	QoL/14 days preoperative, 14 days postoperative, and 6 months postoperative/SF-36	Yes*	Any complications/6 months postsurgery	7
Bloemen	2009	The Netherlands	Primary	N=121/170 Nc=33	Patients with rectal cancer	Observational, cross-sectional	Surgical treatment for adenocarcinoma of the rectum	Only severe complications were considered: grade III or IV complications (according to Dindo's model) were defined as severe, whereas absence of complications or grade I and II complications were defined as absent or mild complications/patient records	QoL/36 (16–51) months postoperative/EORTC QLQ-C30 and CR38	Yes	Severe postoperative complications/median of 36 (range 16–51) months postsurgery	6
Bruns	2010	Germany	Secondary	N=96/188 Nc(any morbidity)=30 Nc(wound infections)=10	Patients who underwent curative hepatic resection for malignant or non-malignant diseases, disease free at time of assessment	Observational, cross-sectional	Hepatectomy	Surgical (eg, bile leak or biloma, pneumothorax, wound infection, liver abscess, bleeding, and surgical dehiscence) and medical (eg, pleural effusion, renal failure, hepatic failure, pneumonia, cardiac insufficiency and cholangitis)/patient records	QoL/ 3–36 months postoperative /SF-12	Yes	Wound infections/3–36 months postsurgery	5
Champault	2006	France	Secondary	Nt1=152/? Nt(4)=139 Nc=(unclear)	Consecutive patients operated on for morbid obesity	Observational, cohort, prospective	Laparoscopic placement of a gastric band	Retrospective list: pulmonary atelectasis or pneumonia, prolonged ileus, minor wounds problems and urinary retention. Slippage with a peak incidence during the second postoperative year. Band erosion with penetration into the stomach. Access port problems (infection, haematoma, leak, disconnection), bands explanted, associated with erosion, obstruction, immediate intolerance and recurrent tubing break/method not specified	QoL/preoperative, 1, 3 months and 2 years postoperative/GIQLI	Confounding*	Band removal for complications such as erosion, slippage, intolerance/2-year postsurgery	6
Chang	2010	Taiwan	Secondary	N=102/218 Nc(anastomotic stricture)=12 Nc(gastrojejunal anastomotic ulcer)=9 Nc(upper gastrointestinal bleeding)=1 N(GORD)=2	Patients undergoing bariatric surgery.	Observational, case–control, longitudinal	Roux-en-Y bypass	Operation-related complications, including gastrojejunal anastomotic stricture, gastrojejunal anastomotic ulcer, upper gastrointestinal bleeding and Gastro-oesophageal reflux disease (GORD)/method not specified	QoL/preoperative, 1, 3, 6 and 12 months postoperative/WHOQoL-BREF	Yes*	Any complications/1, 3, 6, 12 months postsurgery	5
Dasgupta	2008	UK	Secondary	Nt1=102/122 Nt2=87 Nt3=80 Nt4=33 Nc=44	Consecutive, patients undergoing liver surgery for liver cancer	Observational, prospective, cohort	Liver resection for hepatic malignancies	Major complications were defined as those associated with systemic illness requiring transfer to a higher level of care (high dependency or intensive care unit) or requiring relaparotomy, or complications needing interventional radiology/method not specified	QoL/preoperative, 6, 12, 36–48 months postoperative/EORTC QLQ-C30	No*	NA	6
Delaney	2003	USA	Secondary	Nt1=109/109 Nt2=82/109 Nc(any)=19 Nc(major)=9	Patients with Crohn's disease	Observational, cohort, prospective	Surgery for CD (abdominal perineal, loop or end stoma)	Retrospectively listed complications: anastomotic leak, intra-abdominal abscess, bleeding, venous thrombosis, renal failure, and pneumonia, dehydration, intra-abdominal abscess, small bowel obstruction and wound infection/database review	QoL/preoperative and 30 days postoperative/CGQL	Yes*	Any complications/30 days postoperative	7
Douma	2011	The Netherlands	Secondary	N=296/? Nc=?	296 patients with FAP who had been surgically treated	Observational, cross-sectional	Surgery for FAP	Surgery-related complications/self-reports+medical records	QoL/0 to >10 years postoperative/SF-36, EORTC-QLQ-C38,Social Functioning subscale of the Dutch version of IBDQ	Yes	Any complications/0 to >10 years postsurgery	2
Dubernard	2006	France	Secondary	Nt1=58/? Nt2=58 Nc=9	Women with colorectal endometriosis who underwent a segmental colorectal resection	Observational, cohort, prospective	Laparoscopic segmental colorectal resection for endometriosis	Retrospectively listed complications: rectovaginal fistulae, vessel injury of the protective colostomy treated by laparoscopic coagulation, uroperitoneum requiring a ureteral stent for 6 weeks and an abscess behind colorectal anastomosis requiring a laparoscopic drainage/patient observations	QoL/preoperative and postoperative/SF-36	No*	NA	6
El-Awady	2009	Egypt	Secondary	N=40/? Nc=14	Patients with inguinal hernia	Observational, prospective, cohort	Anterior open Lichtenstein tension-free hernioplasty	Postoperative complications: seroma, haematoma, secondary infection, neuralgia and anaesthesia/patient observations	QoL/preoperative, 3, 6 and 12 months postoperative/SF-36	No	NA	4
Hawn	2006	USA	Primary	Nt1=1983/3518 Nt2=1526 (77%) Nt3=1603 (81%) Nc(neuralgia t1)=94 Nc(haematoma t1)=51 Nc(orchitis t1)=13 Nc(recurrence t1)=76 Nc(other t1)=124 Nc(neuralgia t2)=105 Nc(haematoma t2)=55 Nc(orchitis t2)=18 Nc(other t2)=150	Men who received a hernia repair	Observational, cohort, prospective	Inguinal herniorrhaphy	Complications were summarised by 4 categories: (1) haematoma/seroma, (2) orchitis, (3) neuralgia of the leg or groin, and (4) other. complications classified as ‘other’ included (1) early postoperative complications (urinary tract infection, urinary retention, and haematuria); (2) life-threatening complications (respiratory insufficiency, myocardial ischaemia, cardiac arrhythmia, intraoperative hypotension and stroke); and (3) long-term complications (4 weeks or more postoperative)/patient reports for neuralgia and orchitis + expert consensus for life-threatening complications	QOL/pre-op, 1 &2 years post-op/SF-36	Yes*	Neuralgia, orchitis/2 years postsurgery	8
Ince	2011	USA	Secondary	Nt1=?/568 Nt2=166 Nc=?	Patients who underwent colorectal resection for benign and malignant diseases.	Observational, cohort, retrospective	Laparoscopic colorectal resection	No reference	QOL/pre-op, 4 weeks post-op/SF-36	No*	NA	3
Kalliomaki	2009	Sweden	Primary	N(total)=184/423 N1=92 (cases) N2=92 (controls)	Patients who had been operated on for groin hernia. Controls matched for age, gender and method of surgical repair were allotted from the group of persons without persisting pain (grade 1 in IPQ)	Observational, case–control, cross-sectional	Hernia repair	Persistent postoperative pain (patients with pain of grade 3, ie, pain that could not be ignored but did not interfere with everyday activities, or higher on IPQ)/patient reports (IPQ) and clinical examination	QoL, anxiety, depression/(on average 4.9 years postoperative, range > 7 years)/SF-36, HADS	Yes	Persistent postoperative/mean of 4.9 years postsurgery	5
Kement	2011	Turkey	Primary	N=253/351 N(incontinence)=28 N(severe incontinence)=9 N(mild incontinence)=19	Consecutive patients with chronic anal fissure who underwent open lateral internal sphincterotomy (LIS).	Observational, cross-sectional	Open lateral internal sphincterotomy	Anal incontinence/patient reports: WIS system + clinical examination	QoL/23.3±7.1 months postoperative/SF-36	Yes	Severe incontinence/23.3 (SD±7.1) months postsurgery	5
Lim	2006	UK	Primary	N=92/112 Nc(leaks)=23 Nc(clinical leaks)=13 Nc(subclinical leaks)=10	Consecutive patients under the care of three consultant surgeons who underwent procedures with LRA	Observational, cross-sectional	LRA	Anastomotic leaks (clinical and subclinical)/patient observations, CT scans, Wireless Capsule Endoscopy (WCE)	QoL/10–18 months postoperative/EORTC QoL	Confounding	Anastomotic leaks/10–18 months postoperative	5
Liu	2010	US	Primary	N=679/1308 Nc(early comps/anastomosis)=54 Nc(late comps/anastomosis)=126 Nc(early comps/anastomosis/rectal cancer only)=42 Nc(late comps/ostomy/rectal cancer only)=105	Patients with long-term colorectal cancer	Observational, cross-sectional	Colorectal cancer surgery	Digestive, skin, genitourinary, surgical, medical, immediate indirect complicationsEarly complications: those that were first recorded within 30 days of the surgery. Late complications: occurring 31 days after surgery/patient computerised data	QoL/ 5–15 years postoperative/modified City of Hope (mCOH)-QoL-Ostomy	Yes	Enterocutaneous fistula for all patients and any late complications for ostomy patients >5 years postsurgery	6
Mentes	2006	Turkey	Primary	Nt1=253/302 Nt2=244 Nc(anal fistula/abscess)=3 Nc(Fecal Incontinence Severity Index (FISI)>0)=7 Nc(FISI, 0 to >4, 21, 7)=3	Patients who underwent lateral internal sphincterotomy (LIS) for CAF	Observational, cohort, prospective	Lateral internal sphincterotomy (LIS) for CAF	Anal incontinence/atient examination+ FISI score	QoL/preoperative (admission) and 12 months postoperative/GIQLI and FIQL	Unclear (due to small number of patients with complications)	NA	6
Pittman	2008	USA	Primary	N=239/322 Nc=56	Veterans with an ostomy after major gastrointestinal surgery requiring an intestinal stoma	Observational, case–control, cross-sectional	Gastrointestinal surgery requiring an intestinal stoma	Ostomy complications: skin problems, leakage and difficulty with adjustment (ie, leakage, peristomal irritant dermitis, pain, bleeding, stomal necrosis, prolapse, stenosis, herniation, retraction, infection, mucotaneous separation, difficulty adjusting)/patient reports	QoL/6 months postoperative/mCOH-QoL-Ostomy	Yes	Ostomy complications (skin problems, leakage)/ 6 months postsurgery	6
Polese	2012	Italy	Primary	N=147/211 Nc(anastomotic stenoses)=22	Patients who underwent elective left colonic or rectal resection and colorectal anastomosis for neoplastic or inflammatory disease	Observational, cross-sectional	Left colonic or rectal resection and colorectal anastomosis	Anastomotic stenosis/clinical examination	QoL/mean 58 (SD±31) months postoperative/SF-36	Yes	Anastomotic stenosis/58 (SD±31) months postsurgery	6
Rea	2007	USA	Primary	Nt1=505/? Nt2=237 Nt3=106 Nc(t2)=41 Nc(t3)=23	Patients who underwent Roux-en-Y gastric bypass (LRYGB) by one surgeon for morbid obesity	Observational, cohort, prospective	LRYGB for morbid obesity without conversion to an open procedure	Postoperative complications requiring intervention/method not specified	QoL/baseline, 1 and 2 years postoperative/SF-36	Yes*	Complications requiring intervention/1 and 2 years postsurgery	6
Riss	2011	Austria	Primary	N1=16/36 (cases) N2=16/? (controls)	Cases: patients operated for rectal cancer and developed anastomotic leak. Controls: patients operated for rectal cancer at the same time period and had an uneventful postoperative course matched by sex, age (±5 years), type of resection, and neoadjuvant therapy	Observational, case–control, cross-sectional	Rectal resection for malignancies on overall pelvic organ function	Anastomotic leakage: defined as grade A (no change in patient's management), grade B (requires active therapeutic intervention but is managed without relaparotomy) and grade C (requires relaparotomy)/review of the institutional colorectal database and individual chart reviews	QoL/106.8 months postoperative (32.4–170.4)/SF-12	No	NA	7
Rutegard	2008	Sweden	Secondary	N=355/446 (79·6%) Nc=56	Patients diagnosed with an oesophageal or cardia cancer who underwent macroscopically and microscopically radical resection	Observational, cross-sectional	Oesophageal resection	Technical surgical complications, including postoperative bleed exceeding 2000 ml or requiring a reoperation, anastomotic insufficiency, necrosis of the substitute, damage to the recurrent nerve, thoracic duct damage or gastric perforation/prospective scrutiny of medical and histopathological records, operation charts, extensive study protocol with predefined exposure alternatives	QoL/6 months postoperative/EORT QLQ-C30, and QLQ-OES1812	Yes	Technical complications/6 months postsurgery	7
Scarpa	2009	Italy	Secondary	N=47/? Nc=?	Patients admitted for intestinal surgery for Crohn's disease	Observational, cross-sectional	Bowel resection through midline laparotomy or with laparoscopic assistance, end ileostomy, stricturoplasty	Medical and surgical complications and need of reoperation (2 anastomotic leaks, 3 intestinal obstructions, 2 intestinal bleeding, and a wound infection were recorded and two relaparotomies)/method not specified	QoL/3 months postoperative/CGQLI	Confounding	Any complications/3 months postsurgery	3
Sharma	2007	UK	Secondary	Nt1=104/110 Nt2=92 Nc=41	Consecutive patients with newly diagnosed colorectal cancer scheduled for elective open resection in one hospital trust	Observational, cohort, prospective	Elective resection for colorectal cancer	Wound, urinary tract and chest infections, cardiac and respiratory complications, deep venous thrombosis, pulmonary embolism and complications related to anastomotic breakdown/method not specified	QoL, anxiety, depression, positive vs negative affectivity, mood states/preoperative (5–12 days preoperative) and 6–8 weeks postoperative/FACT-C, EuroQOL (EQ-5D), HADS, PANAS, MRS	Yes*	Complications within 30 days of operation/6–8 weeks postsurgery	6
Siassi	2009	Germany	Secondary	Nt1=93/113 Nt2,t3=79 Nc=26	Patients undergoing colorectal surgery for benign and malignant disease	Observational, prospective, cohort	Resection of the sigmoid colon or rectum	Postoperative complications (anastomotic leak, wound infection, delayed food intake, fever, and bladder dysfunction)/method not specified	QoL/preoperative, 3 and 12 months postoperative/SF-36 and GLQI	Yes*	Any complications/3 months postsurgery	7
Targarona	2004	Spain	Primary	N=37/46 Nc(recurrent hernias)=3	Patients diagnosed with paraoesophageal or mixed hiatal hernia (types II, III and IV) with >50% of the stomach in the chest	Observational, cross-sectional	Laparoscopic repair of paraoesophageal hiatal hernia	Hernia recurrence (any migration of the cardia to chest level or evidence of a new paraoesophageal sac)/a barium swallow was given to all patients to rule out an anatomic recurrence. An independent radiologist evaluated all the explorations	QoL/≥6 months postoperative (median, 24; range, 6–50)/SF-36, GDSS and GIQLI	Yes	Clinically recurrent hernias/≥6 months postsurgery	5
Viklund	2005	Sweden	Secondary	N=100/146 Nc=44	Patients newly diagnosed with a histologically verified adenocarcinoma or squamous-cell carcinoma of the oesophagus or adenocarcinoma of the gastric cardia that underwent macroscopically and microscopically radical tumour resection	Observational, cross-sectional	Oesophageal resection surgery for cancer	Anastomotic leakage, infections, respiratory insufficiency, cardiac complications, technical complications, anastomotic strictures, and others (intervention needed to treat embolus, deep venous thrombosis, rupture of the wound, intestinal obstruction, stroke, renal failure, or liver failure)/patient records	QoL/6 months postdischarge/QLQ-C30 and OES-24	Yes	Any complications, anastomotic leakage, infection, respiratory insufficiency, cardiac complications, technical complications/6 months postdischarge	7

Symptoms specific to oesophageal cancer.

*Study controlled for patients’ preoperative well-being.

CAF, chronic anal fissure; CGQL, Cleveland Global Quality of Life; COH-QoL Ostomy, City of Hope Quality of Life for Ostomates questionnaire; EORTC, European Organisation for Research and Treatment of Cancer core; EORTC, European Organisation for Research and Treatment of colorectal cancer; FACT-C, Functional Assessment of Cancer Therapy questionnaire with the colorectal module; FAP, familial adenomatous polyposis; FIQL, Fecal Incontinence Quality of Life Instrument; GDSS, Glasgow Dyspepsia Severity Score; GIQLI, Gastrointestinal Quality of Life Index; GLQI, Gastrointestinal Quality of Life Index; HADS, Hospital Anxiety and Depression Scale; IBDQ, Inflammatory Bowel Disease Questionnaire; IPQ, Inguinal Pain Questionnaire; LRA, low rectal anastomosis; MRS, Mood Rating Scale; NA, not available; OES, Oesophageal Cancer-Specific questionnaire; PANAS, positive and negative affect schedule; SF, Short Form Health Survey; WHOQoL BREF, WHO Quality of Life—Brief; WIS, Wexner Incontinence Score.

**Table 2:** Key characteristics of cardio-thoracic surgery studies (n=17)

First author name	Year	Country	Primary or secondary aim	Sample (N=number of patients in analysis/eligible patients, Nt(i)=sample size per time point, Nc=patients with complications, N1=cases vs N2=controls)	Patient inclusion criteria	Study design	Type of surgery	Surgical complications/method of recording	Psychosocial outcome/time points/measurement tool	Significant association of complications with well-being (yes/no/confounding)	Types of complications and time points of significant effects	Quality assessment score (out of 8)
Deaton	2009	USA	Secondary	Nt1=317/442 Nt2=270 Nc=44% (130)	Patients with documented T2DM undergoing CABG	Observational, cohort, prospective	CABG	Infection of the leg, thorax, sternum, bloodstream or urinary tract; central neurological deficit (stroke or transient ischemia, coma); pneumonia, pulmonary insufficiency with prolonged ventilation or reintubation, pulmonary embolism; renal failure; arrhythmias requiring treatment; prolonged inotropic support or use of intra-aortic balloon pump; or reoperation for bleeding or tamponade/patient records	QoL/3 months post-op/SF-36	Yes	Any complications/3 months postsurgery	6
El Baz	2008	The Netherlands	Secondary	Nt1=198/256 Nt2=168 Nc=?	Consecutive patients who were scheduled for CABG following a coronary angiography	Observational, cohort, prospective	CABG	Postoperative events such as use of inotropes, atrial arrhythmias, or ventricular arrhythmias, sternal resuturing, re-exploration for bleeding, and time spent on mechanical ventilation/registry database, medical notes, outpatient notes and intensive therapy unit charts	QoL/preoperative and 6 months postoperative/SF-36	Yes*	Re-exploration for bleeding and sternal resuturing/6 months postsurgery	8
Ferguson	2009	USA	Primary	N=124/221 Nc=22	Prospective patients who underwent major lung resection for early stage lung cancer.	Observational, cross-sectional	Major lung resection for early stage lung cancer (lobectomy, bilobectomy, pneumonectomy)	Complications were categorised as pulmonary (pneumonia, prolonged intubation, reintubation, air leak more than 7 days, lobar collapse requiring intervention), cardiovascular (pulmonary embolism, myocardial infarction, new postoperative arrhythmia, need for intravenous inotropic agents), other, and any complication/administrative database, hospital medical records, office shadow files	QoL/average of 2.6 years postoperative (3 months to 6.4 years)/EORTC QLQ-C30, EORTC QLQLC13 and DASS-21	Yes	Pulmonary complications/2.6 years postsurgery (range 3 months to 6.4 years)	6
Gjeilo	2010	Norway	Primary	Nt1=534/631 Nt2=462 Nt3=465 Nc(t2)=52	Patients undergoing cardiac surgery	Observational, cohort, prospective	Midline sternotomy	Chronic pain (pain arising after surgery and persisting either continuously or intermittently for 3 months or more/BPI	QoL/preoperative, 6 and 12 months postoperative/SF-36	Yes*	Chronic postsurgical pain/12 months postsurgery	6
Hata	2006	Japan	Secondary	N=452/452 Nc=?	Consecutive adult patients who underwent open heart surgery	Observational, cross-sectional	CABG	Postoperative morbidity (minor stroke, infection, pneumonia, haemodialysis, paraplesis)/patient records	Depression/5–7 days postopertive/interviewed by a psychiatrist and CES-D	Confounding	Postoperative minor stroke and pneumonia/5–7 days postsurgery	6
Jarvinen	2004	Finland	Primary	Nt1=501/1128 Nt2=485 Nc=80	Patients who underwent CABG	Observational, cohort, prospective	CABG (89% via sternotomy incision with cardiopulmonary bypass (CPB; on-pump) and 11% without CPB (off-pump))	Perioperative myocardial infarctions/clinical examination + clinical tests (ECGs, echocardiography, laboratory tests)	QoL/preoperative and 12 months postoperative/RAND-36	Yes*	Perioperative myocardial infarctions /12 months postsurgery	7
Jideus	2009	Sweden	Primary	N1=73/84 (cases) N2=42/? (controls)	Cases: patients who developed SWI after cardiopulmonary bypass Controls: patients prior to CABG and evaluated 1 year postoperative and matched for time of the operation, age and sex	Observational, case–control, cross-sectional	Cardiopulmonary bypass	SWIs: deep infection involving retrosternal tissue and/or the sternal bone)/clinical examination	QoL/20 months postoperative (range 7–40)/SF-36	Yes*	Serious wound infections/20 (range 7–40) months postsurgery	4
Kinney	2012	USA	Primary	N=99 Nt1=120/? Nt2=99 Nc=75	Patients aged 45–75 years undergoing elective thoracotomy	Observational, cohort, prospective	Serratus-sparing posterolateral thoracotomy or limited thoracotomy	Chronic post-thoracotomy pain/Leeds Assessment of Neuropathic Symptoms and Signs + self-reports	QoL/preoperative, 3 months postoperative/SF-36	Yes*	Chronic post-thoracotomy pain/3 months postsurgery	7
Landoni	2006	Italy	Primary	N1=22/42 (cases) N2=40/42 (controls)	Cases: patients who underwent cardiac surgery and developed ARF requiring RRT and left the hospital aliveControls: matched controls who did not develop ARF and did not receive RRT	Observational, case–control, cross-sectional	Cardiac surgery (procedures not specified)	ARF requiring RRT/administrative database, registry	QoL/23–42 months post-op/SF-36	No	NA	6
Le Grande	2006	Australia	Secondary	Nt1=182/444 Nt2=128 Nt3=114 Nc=?	Adults on the waiting list for CABG	Observational, cohort, prospective	CABG	Postsurgical complications such as cardiac arrhythmias, stroke and infections/medical records	QoL/preoperative, 2 and 6 months postoperative/SF-36	Yes*	New cardiac arrhythmia postsurgery, atrial fibrillation/6 months postsurgery	7
Martin	2008	USA	Primary	Nt1=836/2,007 Nt2=2.007 Nc=189	Patients undergoing elective open heart surgery	Observational, cohort, prospective	Open heart surgery (133 valve procedure; 620 CABG; 67 CABG plus valve procedure; 15 CABG plus other cardiac procedure; and 1 closure of an atrial septal defect)	Perioperative myocardial infarction, mediastinitis, superficial wound infection, septicaemia, permanent stroke, transient ischaemic attack, continuous coma, prolonged intubation, ventilator-associated pneumonia, cardiac tamponade, atrial fibrillation, reoperation for bleeding, renal failure, renal failure which required dialysis, and length of stay/method not specified	QoL/preopeative, 1 year postopeative/SF-20	No*	NA	6
Merkouris	2009	Greece	Secondary	Nt1=63/63 Nt2=59 Nt3=56 Nc=42	All patients over 65 presenting a 1, 2 or 3 vessel disease treated with CABG without concurrent procedures (eg, valve replacement)	Observational, cohort, prospective	CABG	Retrospective list of complications: atrial fibrillation, re-exploration for bleeding, low cardiac output syndrome, acute respiratory failure, sternal wound infection, neurological dysfunction, mild problems related to leg incision healing or swelling, chest incision discomfort and medications/method not specified	QoL/preopeative, 4 and 12 months postopeative/MacNew Heart Disease HRQoL questionnaire	No*	NA	5
Moller	2012	Sweden	Secondary	Nt1=249/? Nt2=213 Nc=?	Prospective patients scheduled for lung surgery for lung cancer	Observational, cohort, prospective	Lung surgery	Complication was defined as any of the following postoperative complications: new onset atrial fibrillation, prolonged air leak (chest tubes in place for more than 5 days), pneumonia, reintubation, reoperation, or hospital stay of 8 days or more/method not specified	QoL/preoperative, 6 months postoperative/SF-36	Yes*	Any complications/6 months postsurgery	6
Myles	2001 and 2006	Australia	Secondary	Nt1=120/125 Nt2=120 (days 1, 2, 3) Nt3=108 Nt4=94 Nc=69	Adult cardiac surgical patients	Observational, cohort, prospective	Cardiac surgery (specific procedures not specified)	Respiratory: postoperative mechanical ventilation for more than 24 h or pneumonia, defined as pulmonary infiltrate with positive microbial cultures Cardiac: arrhythmia requiring treatment with antiarrhythmic medication or electrical cardioversion reversion; radiological evidence of pulmonary oedema; or myocardial infarction, defined by new Q waves on ECG or creatine kinase-MB isoenzyme concentration greater than twice normal Renal: acute renal failure, defined by serum creatinine concentration greater than 200 M Neurological: stroke, defined as a new central neurological deficit Sepsis: wound infection requiring excision of tissue or antibiotic therapy, or positive microbial culture (other than pneumonia) Clinical and laboratory tests (microbial cultures, radiological data, ECGs, etc)	QoL/preoperative, 1 and 3 months, 3 years postoperative/SF-36	Confounding*	Any complications/3 months postsurgery	8
Peric	2008	Serbia and Montenegro	Secondary	Nt1=208/? Nt2=192 Nc=60	Consecutive patients who underwent elective CABG	Observational, cohort, prospective	CABG	Retrospective list of complications: low cardiac output (cardiac index lower than 2 L/min/m^2^), mechanical ventilation longer than 24 h, reoperation for bleeding, sternal wound infection, perioperative myocardial infarction, pericardial effusion, arrhythmic complications (atrial fibrillation, ventricular tachycardia, ventricular fibrillation), abdominal complications, and other/observations, ECGs, echocardiography, laboratory tests	QoL/preoperative, 6 months postoperative/NHP Questionnaire	Yes*	Any complications/6 months postsurgery	7
Rodriguez	2008	USA	Secondary	Nt1=397/? Nt2=? Nt3=? Nt4=? Nc=23	Patients diagnosed with upper extremity hyperhidrosis (HH) treated with thoracic sympathectomy (TS)	Observational, cohort, prospective	Thoracoscopic sympathectomy for palmar and axillary hyperhidrosis	CS: excessive sweating considered abnormal in other parts of the body after TS Gustatory sweating: facial sweating after eating foods Excessive dryness: dryness affecting the hands and requiring hydration Method not specified	QoL/preoperative, discharge, 6 and 12 months postoperative/SF-36	No*	NA	3
Tully	2011	Australia	Primary	Nt1=226/238 Nt2=222 Nc=56	Patients undergoing first-time CABG surgery	Observational, cohort, prospective	CABG	New-onset AF between the patient's day of admission to the intensive care unit and the median day of discharge (day 5) after CABG during the index hospitalisation/ECGs, transthoracic echocardiographs reviewed by technicians and reviewers blinded to patients’ psychological distress scores	Anxiety, depression, stress/preoperative (mean=2 days, SD=2 days) and postoperative (mean=6 days, SD=2 days)/ DASS	Yes*	Atrial fibrillation/6 days (SD=2 days) postsurgery	7

*Study controlled for patients’ preoperative well-being.

ARF, acute renal failure; AF, atrial fibrillation; BPI, Brief Pain Inventory; CES-D, Center for Epidemiological Studies Depression Scale; CS, compensatory sweating; DASS, Depression Anxiety Stress Scales; DASS, Short version of the Depression Anxiety Stress Scales; EORTC QLQLC, European Organisation for Research and Treatment of Cancer core Lung Cancer Questionnaire; HRQoL, health-related quality of life; NA, not available; NHP, Nottingham Health Profile; QoL, quality of life; RRT, renal replacement therapy; SF, Short Form Health Survey; SWI, sternal wound infection; T2DM, type 2 diabetes mellitus.

**Table 3:** Key characteristics of studies in vascular surgery (n=4)

First author name	Year	Country	Primary or secondary aim	Sample (N=number of patients in analysis/eligible patients, Nt(i)=sample size per time point, Nc=patients with complications, N1=cases vs N2=controls)	Patient inclusion criteria	Study design	Type of surgery	Surgical complications/method of recording	Psychosocial outcome and time points	Significant association of complications with well-being (Yes/No/Confounding)	Types of complications and time-points of significant effects	Quality assessment score (out of 8)
Lohse	2009	Germany	Secondary	N=110/124 Nc=?	Consecutive patients who received a replacement of the dilated ascending aorta	Observational, cross-sectional	Ascending aorta replacement	Retrospective list: postoperative bleeding, myocardial infarction, stroke, pneumonia, respiratory insufficiency, acute renal dysfunction, sepsis, lung fistula/method not specified	QoL/36.4±15.5 months postoperative (11–58 months)/SF-36	NO	NA	**4**
Nguyen^a^	2007	USA and Canada	Primary	Nt1=1296/1404 Nt2=862 Nt3=732 Nc=543	Patients who underwent lower extremity vein bypass for CLI in community and university hospitals across the US and Canada	Observational, cohort, prospective	Lower extremity vein bypass for limb salvage in CLI patients	Wound complications (WC): patients having infection, necrosis, hematoma-haemorrhage, or seroma-lymphocele at the surgical incision or harvest site within 30 days of the bypass surgery/Adverse events clinical trial documentation with reference to source documentation (hospital notes etc.)	QoL/baseline, 3 and 12 months postoperative/VascuQol	Confounding*	Wound complications/3 months postsurgery	**8**
Nguyen^b^	2006	USA and Canada	Secondary	N1=1296/1404 (92.3%) N2=862 (61.4%) N3=732 (52.1%) Nc=?	Patients who underwent IB for CLI in community and university hospitals across the USA and Canada	Observational, cohort, prospective	Infrainguinal vein grafting for limb salvage in patients with CLI	GREs: development of a >70% graft stenosis or having undergone a percutaneous or surgical revision or a major amputation/clinical tests (angiography, ultrasonography, etc), source documentation (hospital notes, discharge notes, operative and procedural notes, etc)	QoL/preoperative, 3 and 12 months postoperative/VascuQol	Yes*	GREs/12 months postsurgery	**8**
Subramonia	2005	UK	Primary	Nt1=70/70 Nt2=59 Nt3=62 Nc(sensory abnormalities)=25 Nc(bruising at t1)=58 Nc(bruising at t2)=16	Patients with varicose veins, either symptomatic or with skin changes, resulting from incompetence of the lesser saphenous vein system (LSV) as confirmed by handheld Doppler examination or duplex ultrasonography or both and requiring surgical intervention (both day cases and inpatients)	Observational, cohort, prospective	Conventional LSV stripping	Bruising/tracing method Sensory abnormalities, both subjective (paraesthesia and dysaesthesia) and objective/patient reports, sensory testing	QoL/preoperative, discharge and 6 weeks postoperative/Aberdeen Varicose Vein Questionnaire 2	No*	NA	**7**

*Study controlled for patients’ preoperative well-being.

CLI, critical limb ischaemia; GRE, graft-related event; NA, not available; QoL, quality of life; VascuQol, a validated instrument assessing pain, symptoms, activities, social life and emotional state in patients with vascular disease.

QoL was the main reported psychosocial outcome. Three studies measured anxiety,^30^ ^40^ ^62^ four studies measured depression^31^ ^41^ ^49^ ^62^ and one study measured mood states.^41^ No other psychosocial outcomes were measured. The Short Form Health Survey (SF)-36 (and its associated versions, ie, SF-12, SF-20) was the most commonly used scale for the measurement of QoL.^18^ ^25–31^ ^36–38^ ^42^ ^43^ ^45^ ^46^ ^48^ ^51–55^ ^57–59^ ^61^ ^63^

The vast majority of the studies used a priori definitions of complications. For example, Bloemen *et al*^19^ recorded only severe complications based on a grading system of surgical complications. Dasgupta *et al*^23^ also recorded major complications which were defined as “those associated with systemic illness requiring transfer to a higher level of care or requiring relaparotomy, or complications needing interventional radiology”. Others used predefined categories of complications such as infections, respiratory complications, chronic postoperative pain or perioperative myocardial infarctions. A total of 14 studies did not define or describe the complications that were recorded. The majority of the studies recorded a range of postoperative complications. Eighteen studies focused on a single category of complications (eg, anastomotic leaks, perioperative myocardial infarctions, wound complications, atrial fibrillation). Complications were mostly recorded through medical records review, clinical examinations and review of administrative databases.

Study quality varied. The scores of the included studies ranged from 2 to 8, with a mean score of 5.9. Points were deducted for the following reasons: lack of information on how complications were defined or on the methods for their recording,^16–18^ ^21–23^ ^25^ ^29^ ^35^ ^37^ ^40–42^ ^46^ ^51^ ^55–57^ ^61^ ^63^ lack of information on response rates,^16^ ^21^ ^22^ ^25–27^ ^29^ ^37^ ^40^ ^50^ ^52^ ^54^ ^55^ ^57^ ^60^ ^61^ baseline psychosocial outcomes were either not measured or controlled for,^17^ ^19^ ^20^ ^25^ ^27^ ^30–36^ ^38–40^ ^43–45^ ^47^ ^49^ ^53^ ^63^ and demographic or clinical factors were not controlled for.^20^ ^25^ ^27^ ^31^ ^32^ ^34^ ^40^ ^43^ ^45^ ^51^ ^56^ ^61^ ^63^ Seven studies scored exceptionally low (ie, below 4).

### The impact of surgical complications on patients’ well-being

The majority of studies (n=32) found that patients who suffered surgical complications had significantly worse postoperative psychosocial outcomes than patients with uncomplicated recovery.^16–20^ ^22^ ^24^ ^25^ ^28^ ^30^ ^31^ ^33^ ^35–37^ ^39^ ^41–48^ ^50–52^ ^54^ ^57^ ^60^ ^62^ ^65^ This was the case not only after major surgical procedures but also after relatively minor operations such as hernia repairs.^18^ ^28^ ^30^ ^31^ ^43^ The vast majority (n=25, 78%) were of high quality (ie, quality assessment score greater than 6 out of 8). For instance, more than half of the studies with significant findings had measured and controlled for patients’ baseline psychosocial outcomes (n=18)^16^ ^18^ ^22^ ^24^ ^28^ ^37^ ^41^ ^42^ ^46^ ^48^ ^50–52^ ^54^ ^57^ ^60^ ^62^ ^65^ and used multivariate analyses (n=21),^16^ ^18^ ^19^ ^22^ ^24^ ^25^ ^28^ ^35^ ^37^ ^39^ ^41^ ^42^ ^44^ ^46^ ^47^ ^50^ ^52^ ^54^ ^60^ ^62^ ^65^ suggesting that complications remained a significant independent predictor of patients’ postoperative well-being even after controlling for a range of clinical and demographic factors. Psychosocial outcomes that were significantly negatively affected by surgical complications included physical, emotional and social aspects of patients’ QoL as well as anxiety and depression levels (see table 4). Complications that were found to be significantly associated with worse psychosocial outcomes included both major events such as perioperative myocardial infarctions after CABG,^50^ severe incontinence after internal sphincterectomy^31^ or graft-related events after vascular surgery,^65^ and minor complications such as wound infections after hepatic resection,^20^ or new cardiac arrhythmias after CABG.^54^ The complications that were significantly associated with patients’ postoperative psychosocial outcomes are presented in tables 1–3.

**Table 4:** Domains of patients’ well-being that were significantly affected by surgical complications

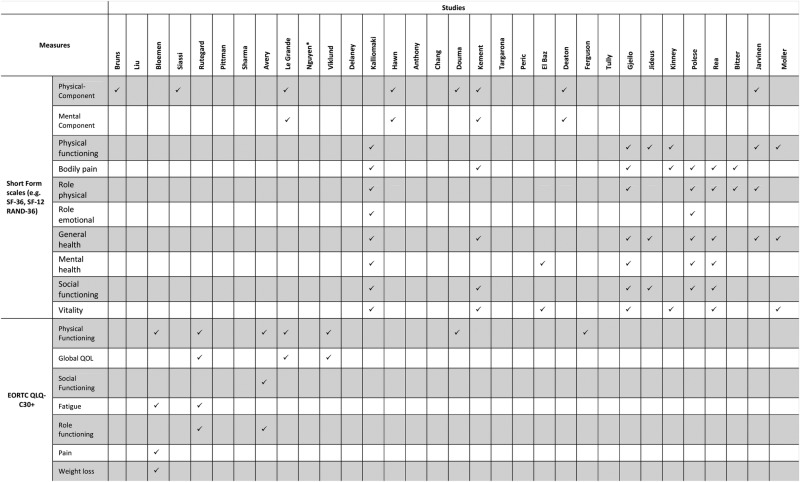																																

Six studies reported a confounding association between surgical complications and patients’ well-being (ie, complications were significantly associated with worse psychosocial outcomes only under certain conditions)^21^ ^32^ ^40^ or complications were significantly associated with psychosocial outcomes at univariate but not at multivariate analysis.^49^ ^59^ ^64^ A total of 12 studies did not find a significant association of surgical complications with postoperative psychosocial outcomes.^23^ ^26^ ^27^ ^29^ ^34^ ^38^ ^53^ ^55^ ^56^ ^61^ ^63^ ^66^ The majority of them (n=7) scored below 6 on quality assessment. For example, four studies had very small samples.^26^ ^27^ ^34^ ^38^

#### Meta-analyses

A series of supplementary meta-analyses were attempted on each extracted psychosocial outcome (ie, QoL, anxiety, depression). For a meta-analysis on QoL, a synthesis of data from widely disparate assessment tools with very different composite scores (eg, social, emotional and physical) was not considered valid. For that reason, only studies that used the SF scales^67^ were considered as they were the most commonly used QoL measures. Only three studies had sufficient data on the SF physical and mental QoL component scores.^28^ ^31^ ^45^ The pooled mean differences (MD) between the two groups were statistically significant (p<0.001), indicating lower levels of physical (MD=−3.28, CI −4.71 to −1.86) and mental (MD=−3.82, CI −4.97 to −2.67) QoL in patients who suffered complications compared with patients without complications. Two studies provided sufficient data for a meta-analysis on anxiety.^30^ ^62^ The pooled standardised MD was not significant (p>0.05). A meta-analysis on depression was not possible as there was only one study with available data.^30^

For a more detailed report of the meta-analyses, see online supplementary materials 2–4.

### The duration of the impact of surgical complications on patients’ well-being

Eighteen studies which reported significant associations of complications with postoperative psychosocial outcomes found a significant relationship of the presence of postoperative complications with worse psychosocial outcomes at 12 months postsurgery or later.^16^ ^19–22^ ^25^ ^28^ ^30–33^ ^36^ ^37^ ^47^ ^48^ ^50^ ^51^ ^65^ Twenty studies reported a significant association of complications with worse psychosocial outcomes at less than 12 months postsurgery.^17^ ^18^ ^24^ ^35^ ^39–46^ ^49^ ^52^ ^54^ ^57^ ^59^ ^60^ ^62^ ^64^

## Discussion

This is, to our knowledge, the first systematic review of the literature investigating the impact of surgical complications on patients’ psychosocial well-being. In line with our hypothesis, two-thirds of the included studies found a significant negative association between the occurrence of surgical complications and patients’ postoperative well-being. The vast majority of those studies were of high quality. For instance, more than half of the studies with significant findings found that complications were an independent predictor of postoperative psychosocial outcomes after controlling for pre-existing differences on psychosocial outcomes, clinical and demographic variables.

Significant associations were reported in individual studies between surgical complications and lower scores on physical, emotional and social dimensions of the various QoL measures. A meta-analysis of three studies with sufficient QoL data collected with the SF scales suggests significant adverse effects of complications both on the physical and the mental health components. These findings are in agreement with earlier preliminary findings on the psychological burden that surgical adverse events often impose on patients.^3^ ^4^ Surgical complications were also significantly associated with higher postoperative anxiety and depression in individual studies, even though a population effect could not be shown due to the very small number of studies that measured the impact of surgical complications on anxiety and depression. Despite the fact that QoL is a useful screening outcome offering a general picture of a person's physical health and psychological state,^68^ future studies on the psychosocial impact of surgical complications should also consider outcomes such as anxiety and depression as they offer a more accurate picture of a person's psychological well-being. Other relevant psychological outcomes such as post-traumatic stress, which was not measured in any of the included studies, would also be of relevance for future research in this area. It is also worth noting that strong conclusions cannot be drawn on the basis of the meta-analyses results due to the small number of studies included in them.

Complications that were found to significantly contribute to patients’ low postoperative well-being ranged from severe adverse events such as anastomotic leaks after gastrointestinal surgery or perioperative myocardial infarctions after cardiac surgery to relatively minor complications such as wound infections or atrial fibrillation. It appears therefore that other than severe postoperative events, minor complications could also cause psychological distress during patients’ recovery. For instance, wound complications could affect patients’ satisfaction with their body image which could further compromise their QoL and psychological well-being.^69^ This finding potentially implies that the severity of complications as judged by healthcare professionals does not always correspond with patients’ experience of complications. Moreover, complications were negatively associated with postoperative psychosocial outcomes not only after major surgical procedures but also after relatively minor operations,^18^ ^28^ ^30^ ^31^ ^43^ which suggests a potential independence of the magnitude of initial surgery with the effect of complications on patients’ well-being. Further research on how complications affect patients’ well-being after different types of surgery could help clarify this finding.

**Table 4:** Continued

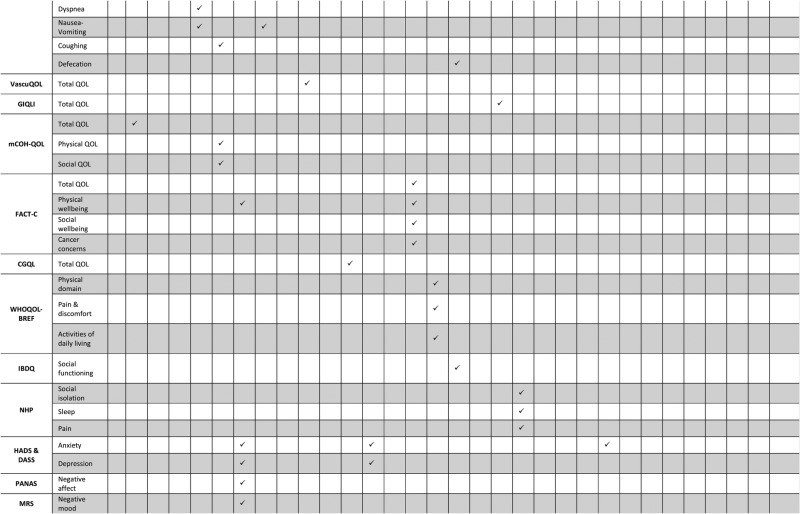																																

CGQL, Cleveland Global Quality of Life; DASS, Depression Anxiety Stress Scales; EORTC, European Organisation for Research and Treatment of Cancer; FACT-C, Functional Assessment of Cancer Therapy questionnaire with the colorectal module; GIQLI, Gastrointestinal Quality of Life Index; HADS, Hospital Anxiety and Depression Scale; IBDQ, Inflammatory Bowel Disease Questionnaire; MRS, Mood Rating Scale; NHP, Nottingham Health Profile; PANAS, positive and negative affect schedule; QoL, quality of life; VascuQoL, a validated instrument assessing pain, symptoms, activities, social life and emotional state in patients with vascular disease.

A number of studies also found a significant negative contribution of surgical complications to psychosocial outcomes more than 1 year postoperatively, suggesting that patients may suffer psychologically due to the experience of surgical complications for an extensive period of time after surgery. The above findings hold important implications for patients’ recovery as there is growing evidence on the role of psychological stress in compromising the function of the immune system and slowing down wound healing.^7–9^ Surgical complications are likely to further prolong patients’ recovery in almost a reciprocal cycle of distress and decreased immune function. The exact relationships between surgical complications, psychological distress and speed of recovery warrant further investigation.

It is noteworthy that a smaller number of studies did not find a significant association between complications and patients’ postoperative psychosocial outcomes or found significant univariate associations which were not replicated in multivariate analyses. Even in studies showing a significant impact, there will be many patients who largely maintain their psychological health and QoL in the aftermath of complications. Other than clinical factors, patients’ ways of coping with stress, their appraisals of surgery and their health, as well as their perceptions of support from their loved ones and healthcare professionals could explain the conditions under which complications affect patients’ well-being, as suggested by wider literature on patients’ adjustment after surgical treatment.^70–72^ The role of psychological factors as potential moderators of the psychological impact of surgical complications needs to be further explored.

Overall, the quality of the included studies was good as indicated by their relatively high-quality assessment scores and the small number of studies that scored exceptionally low. A substantial number of studies with significant findings controlled not only for patients’ preoperative psychosocial outcomes but also for a variety of clinical and demographic factors confirming that surgical complications were an independent predictor of postoperative psychosocial outcomes above and beyond any pre-existing differences. The fact that the included studies used validated self-report measures for the measurement of psychosocial outcomes and the use of a very comprehensive search strategy also increase the validity of the findings.

### Limitations

A few caveats should be borne in mind when interpreting the above findings. First, one-third of the studies did not define complications or did not describe the methods they used to record complications. Moreover, almost one-third of the studies did not provide information on response rates, which does not allow inferences about the representativeness of their samples.

Regarding the methodology of the systematic review, studies that were published before the year 2000 or with the majority of patients recruited before the year 2000 were excluded, albeit limiting this review to literature that was published in the last decade is expected to be more reflective of current surgical practice. It should also be noted that studies that were published past the final run of the search strategy (ie, May 2012) have not been considered. Caution should also be taken when interpreting these findings to other specialties as the clinical setting in which complications occur may affect their impact on patients’ well-being. Another limitation was the very small number of studies with sufficient data for quantitative synthesis and the difficulty of synthesising data from different QoL measures, which resulted in restricting the meta-analyses on data collected only with the SF scales. The small number of studies with available data did not permit certain types of sensitivity analyses such as by surgical specialty, type of surgery (ie, minor vs major surgery) or underlying disease (eg, cancer vs other conditions), which could be significant determinants of the impact of complications on patients’ well-being. Lastly, there is always the potential for publication bias where studies with significant results and big effect sizes are more easily published.^73–75^ It is worth adding that none of the included studies were randomised controlled trials due to the non-appropriateness of this design for the research questions that this review aims to answer.

### Implications of findings

The results highlight the importance of considering patients’ psychological needs in the aftermath of surgical complications. Surgical and nursing staff need to be aware of the challenges of surgical complications for patients’ well-being and ensure that their psychological needs are not neglected. Screening patients who suffer postoperative complications for symptoms of psychological distress could help identify those patients who need psychological support. Facilitating patients’ access to psychological support during and after their hospital stay could also be of great value for patients’ postoperative well-being. For example, early referral to psychological services could prevent long-term psychological distress and may also mitigate the negative effects of stress on patients’ recovery. Primary care practitioners and carers need to be aware of the psychological burden that surgical complications impose on patients in order to recognise their distress in time and to provide the support that patients need.

## Conclusions

This is the first systematic review of the literature on the impact of surgical complications on patients’ psychosocial well-being. The findings of this review suggest that surgical complications are potentially a significant independent predictor of patients’ impaired postoperative psychosocial well-being often for a very long time postsurgery. It also appears that other than major complications, relatively minor adverse events may also compromise patients’ psychosocial well-being, which implies that the clinical severity of complications may not always indicate how seriously patients will be affected by them. Patients who experience surgical complications report worse levels of different aspects of QoL than patients with uncomplicated recovery, often more than a year after their operation. The ways in which complications are managed (eg, reoperation vs conservative management), the type of surgery (eg, minor vs major), the underlying disease (eg, cancer vs other conditions), psychological factors (eg, patients’ perceptions of support, illness perceptions, coping strategies) or cultural influences may be key moderators of the impact of surgical complications on patients’ psychosocial well-being. Future research is needed on the contribution of the above factors on the impact of surgical complications on psychological outcomes such as anxiety, depression and post-traumatic stress, as well as on how to support patients who experience a complicated postoperative recovery.
